# RAGE in Neutrophils: A Sensor for Pathogen-Associated Structures and Beyond

**DOI:** 10.3390/biomedicines14051128

**Published:** 2026-05-16

**Authors:** Ekaterina A. Golenkina, Sofia V. Navarnova, Galina M. Viryasova, Svetlana I. Galkina, Tatjana V. Gaponova, Yulia M. Romanova, Galina F. Sud’ina

**Affiliations:** 1 Belozersky Institute of Physico-Chemical Biology, Lomonosov Moscow State University, 119234 Moscow, Russia; golyesha@mail.ru (E.A.G.); galina.viryasova@yandex.ru (G.M.V.); galkina@genebee.msu.ru (S.I.G.); 2 Faculty of Bioengineering and Bioinformatics, Lomonosov Moscow State University, 119234 Moscow, Russia; navarnova.soffia@gmail.com; 3 National Research Center for Hematology, Russia Federation Ministry of Public Health, 125167 Moscow, Russia; gaponova.tatj@yandex.ru; 4 Gamaleya National Research Centre of Epidemiology and Microbiology, 123098 Moscow, Russia; genes2007@yandex.ru

**Keywords:** neutrophil, *Salmonella typhimurium*, RAGE, soluble RAGE (sRAGE), intracellular calcium, reactive oxygen species, phagocytosis

## Abstract

**Background/Objectives**: Neutrophils express the receptor for advanced glycation end products (RAGE), yet its role in antibacterial responses remains incompletely defined. This study aims to elucidate the dual functionality of RAGE as a membrane-bound signaling sensor and a source of soluble RAGE (sRAGE) in human neutrophils challenged with *Salmonella typhimurium*, a clinically relevant Gram-negative pathogen. **Methods**: Human peripheral neutrophils from healthy donors were isolated and stimulated with *S. typhimurium*, LPS, or fMLP. Calcium flux, ROS/RNS production, and phagocytosis were assessed using fluorescent probes and spectroscopy. RAGE expression and localization were analyzed by immunofluorescence microscopy and flow cytometry. Soluble RAGE in supernatants was quantified by ELISA, and its molecular forms were characterized by Western blotting. **Results**: Resting neutrophils exhibited minimal surface RAGE but a substantial intracellular pool. RAGE inhibition with FPS-ZM1 attenuated bacteria-induced Ca^2+^ mobilization, oxidative burst, nitrosative output, and phagocytosis, with the most pronounced defect at the pathogen-attachment stage—consistent with impaired cytoskeletal remodeling. Upon activation, neutrophils rapidly released sRAGE (peak at ~10 min) via combined metalloprotease-dependent shedding and regulated secretion of pre-formed intracellular stores. Paradoxically, FPS-ZM1 amplified sRAGE release while suppressing membrane-proximal signaling. **Conclusions**: Neutrophil RAGE functions as a dynamic, multi-compartmental regulator: membrane-associated RAGE licenses effector responses to Gram-negative bacteria, while concomitant sRAGE release provides a fast negative-feedback loop to limit excessive inflammation. This self-limiting circuit balances antimicrobial defense with tissue protection, and its dysregulation may contribute to pathological outcomes in acute and chronic infections.

## 1. Introduction

The receptor for advanced glycation end products (RAGE) is a multi-ligand pattern-recognition receptor (PRR) of the immunoglobulin superfamily, structurally related to MHC class I molecules [1]. While RAGE is highly expressed during embryonic development and in certain adult tissues, notably the lung, to support morphogenesis and epithelial homeostasis [2,3,4], its expression in most differentiated cells remains low under physiological conditions [5]. In recent decades, research on the RAGE receptor has primarily focused on its role in chronic inflammatory and degenerative diseases, where the accumulation of ligands, namely advanced glycation end products (AGEs), triggers sustained receptor activation, generating a positive inflammatory feedback loop [6,7,8,9]. However, growing evidence indicates that RAGE functions as a broad sensor in innate immunity, contributing to immune cell maturation, tissue repair, and acute inflammatory responses [10,11]. Despite these advances, a comprehensive understanding of its role in rapidly responding effector cells under physiological conditions remains lacking.

Neutrophils (polymorphonuclear leukocytes, PMNLs), the most numerous innate immune cells and key effectors of host defense, express RAGE [12]. However, the nature of RAGE-mediated signaling in these cells is multifaceted and highly context-dependent. Historically, research on RAGE in neutrophils has focused on diabetic complications, where AGE-RAGE signaling impairs chemotaxis, disrupts intracellular Ca^2+^ processing, and reduces microbial clearance [13,14]. Notably, in the majority of studies investigating RAGE-mediated inflammatory mechanisms, neutrophils are predominantly characterized as donors of RAGE ligands—infection-triggered alarmins, especially HMGB1 and S100-family proteins—rather than as direct cellular targets of RAGE signaling [15]. Consequently, data on the role of RAGE in the control of AGE-independent neutrophil effector functions remain scarce and often contradictory. While some reports indicate that RAGE engagement promotes integrin upregulation, oxidative burst and cytokine amplification [16], others demonstrate suppression of NADPH oxidase activity and diminished bacterial killing [17]. These discrepancies are likely related to the signaling characteristics of different ligands, species differences, and the possibility of dynamic regulation of receptor availability through proteolytic cleavage. Crucially, it remains unknown whether RAGE acts as an independent trigger for early neutrophil activation cascades (e.g., Ca^2+^ mobilization, ROS generation) or primarily as a co-receptor that amplifies canonical PRR signals. Clarifying this mechanistic ambiguity is essential for understanding how RAGE shapes the magnitude and specificity of the acute immune response and for developing targeted strategies to modulate hyperinflammation without compromising host defense.

RAGE-signaling rarely operates in isolation. Rather, it interacts with Toll-like receptors (TLRs), in particular, TLR4, and supports inflammatory signaling such as NF-κB and MAPK pathways [18]. Moreover, RAGE can act as a nucleic-acid “shuttle” that promotes DNA uptake into endosomes and lowers the activation threshold of TLR9 act as a nucleic-acid shuttle, facilitating DNA uptake into endosomes and modulating the activation threshold of intracellular sensors such as TLR9—a pathway highly relevant for both bacterial infections and sterile inflammation [19]. In humans, direct mechanistic data are still more limited than in mouse models, but there are clinically relevant signals: soluble RAGE (sRAGE) is often elevated in sepsis and correlates with its severity [20], and RAGE-targeting strategies show benefit in several experimental infection/sepsis models [21], supporting the idea that RAGE activity matters in vivo, even if the exact neutrophil-specific contribution varies by pathogen and context.

To dissect RAGE-specific contributions without the confounding effects of chronic ligand accumulation, we employed FPS-ZM1, a well-characterized small-molecule inhibitor that selectively blocks ligand binding to the extracellular V-domain of RAGE [22]. Beyond its utility as a research tool, this compound has demonstrated therapeutic potential in several disease models [23,24,25]. Unlike broad-spectrum immunosuppressants, FPS-ZM1 allows for selective assessment of RAGE-dependent signaling pathways in acute cellular responses.

In this study, we investigated RAGE expression and function in human peripheral neutrophils, focusing on its role in regulating intracellular Ca^2+^ mobilization, ROS production, phagocytosis, and cell spreading upon encounter with Gram-negative *Salmonella typhimurium*. Here, we show that pharmacological blockade of RAGE significantly attenuates early effector responses, establishing an AGE-independent role for the receptor as a primary driver of neutrophil activation. We further characterized resting-state receptor localization, confirming minimal surface expression in unstimulated cells alongside a substantial intracellular pool, and demonstrated that acute activation triggers rapid sRAGE release via a dual mechanism of proteolytic shedding and regulated exocytosis. While the functional consequences of this release remain to be fully elucidated, these findings indicate the existence of a potential autoregulatory circuit and position RAGE as a dynamically controlled immune checkpoint rather than a passive signaling amplifier in acute host defense.

## 2. Materials and Methods

### 2.1. Materials

Dextran T-500 was from Pharmacosmos (Holbæk, Denmark). Dulbecco’s phosphate-buffered saline (D-PBS) with magnesium but without calcium, Hank’s balanced salt solution with calcium and magnesium but without Phenol Red and sodium hydrogen carbonate (HBSS), Iron containing superoxide-dismutase (Fe-SOD) from E. coli, Lipopolysaccharides (LPS) from *Salmonella enterica* serotype *typhimurium* (L6511), and N-Formyl-L-methionyl-L-Leucyl-L-Phenylalanine (fMLP), FPS-ZM1 (Calbiochem^®^) were purchased from Sigma (Steinheim, Germany). Fibrinogen from human plasma and 4,5-Diaminofluorescein diacetate (DAF-2 DA) were purchased from Merck KGaA (Darmstadt, Germany). 6-carboxy-2,7 dichlorodihydrofluorescein diacetate (Carboxy-H_2_DCF-DA), Dihydroethidium (DHE) and Fura-2 AM, Anti-RAGE Recombinant Mouse Monoclonal Antibody [2A11] (Invitrogen, Carlsbad, CA, USA, #MA5-48192), Mouse IgG1 Isotype control (MOPC-21) (Invitrogen, #MA1-10407), Goat anti-Mouse IgG Superclonal Recombinant Secondary Antibody, Alexa Fluor 488 (Invitrogen, #A28175), Rabbit anti-Mouse IgG [H+L] Secondary Antibody, HRP (Invitrogen, # 61-6520) and normal goat serum were purchased from Thermo Fisher Scientific (Waltham, MA, USA). Human AGER (Advanced Glycosylation End Product Specific Receptor) ELISA Kit was purchased from ELK Biotechnology (Sugar Land, TX, USA).

Bacteria (strain *S. typhimurium* IE 147) were obtained from the collection of the N.F. Gamaleya National Research Center for Epidemiology and Microbiology (Moscow, Russia). Bacteria were grown in Luria–Bertani broth to a concentration of 1 × 10^9^ colony-forming units (CFU)/mL. Bacteria were opsonized immediately before the experiment in 10% (*v*/*v*) donor serum for 30 min at 37 °C followed by washing with PBS.

### 2.2. Neutrophil Isolation

Human peripheral neutrophils were isolated from the citrate-anticoagulated blood of healthy volunteers by a previously described standard technique [26]. Experimental and the subject consent procedures were approved by the Bioethics Committee of the Lomonosov Moscow State University, Application # 6-h, version 3, Bioethics Commission meeting # 131-d held on 31 May 2021.

Briefly, dextran sedimentation was used for removing erythrocytes. Leukocyte-rich plasma was separated by centrifugation at 400× *g* through Ficoll-Paque (1.077 g/mL) for 30 min at room temperature; granulocytes and erythrocytes settled to the bottom. The remaining erythrocytes were subjected to hypotonic lysis, followed by double washing with PBS. Neutrophils (96–97% purity, as routinely established by Hoechst and Romanowsky–Giemsa staining followed by fluorescence or light microscopy respectively (Supplementary Figure S1), 98–99% viability as established by trypan blue staining) were then suspended at 2 × 10^7^ cells/mL D-PBS containing 1 mg/mL glucose and stored at room temperature.

### 2.3. Intracellular Free Calcium Ion Concentration ([Ca^2+^]_i_) Assessment

Fluorescent dye Fura-2 AM was used to detect changes in intracellular calcium concentration [Ca^2+^]_i_. The manufacturer’s instructions have been partially modified to work with neutrophils. Briefly, isolated PMNLs (10^7^ cells/mL) were incubated with 1 µM Fura-2 AM in D-PBS containing 1 mg/mL glucose for 30 min at 37 °C. Then, cells were pelleted (from here on for cell sedimentation: 200× *g*, 10 min), washed once with PBS, and resuspended in D-PBS containing 1 mg/mL glucose for at least 30 min. Immediately before the experimental procedure, Fura-2-loaded cells were resuspended in pre-warmed HBSS medium supplemented with 0.01 M HEPES (HBSS/HEPES), seeded in fibrinogen-coated 96-well black F-bottom plates (from here on: 30 µg/mL fibrinogen solution in PBS, coating for 1 h at 37 °C, followed by washing with PBS) at a rate of 4 × 10^5^ cells/100 µL per well, and treated according to the experimental design at 37 °C in a humidified 5% CO_2_ atmosphere. Reagent injectors integrated into the reader platform were used for stimuli addition. Changes in fluorescence emitted at 510 nm were measured when exited by both 380 nm (for Ca^2+^-free dye) and 335 nm (for Ca^2+^-bound dye) every 0.6 s. Manipulations were performed on a CLARIOstar multimode microplate reader (BMG Labtech, Cary, NC, USA) and the MARS data analysis software package version 3.30 from BMG Labtech was used to process the data obtained. [Ca^2+^]_i_ shifts were evaluated by changes in the ratio of fluorescence intensities produced by excitation at two wavelengths. Data were quantified using areas under the kinetic curves (AUC) above the baseline.

### 2.4. ROS Assessment

Hydroxyethidium (EOH) fluorescence measuring was used for detection of total superoxide anion (O^2−^) production [27]. PMNLs were suspended in HBSS/HEPES supplemented with 10 µg/mL DHE and divided into fibrinogen-coated wells of 96-well black F-bottom plates (4 × 10^5^ cells/100 µL per well) followed by incubation at 37 °C, in a humidified 5% CO_2_ atmosphere in accordance with experimental protocol. To detect the intracellular O^2−^ pool, Fe-SOD (100 U/mL) was added to the probes. Fluorescence intensity was measured 40 min after adding stimuli using a CLARIOstar microplate reader with excitation and emission wavelengths of 480 and 567 nm, respectively.

Cytoplasmic ROS production was monitored by measuring green fluorescence of H_2_DCF oxidation product, DCF. According to the manufacturer’s protocol, which was partially adapted to work with neutrophils, PMNLs were incubated with 2.5 µM Carboxy-H_2_DCF-DA for 60 min at RT, washed with PBS and resuspended in D-PBS containing 1 mg/mL glucose for at least 30 min before use. Immediately before the experimental procedure, PMNLs were resuspended in HBSS/HEPES, divided into fibrinogen-coated wells of a 96-well black F-bottom plates (4 × 10^5^ cells/100 µL per well) and incubated at 37 °C, in a humidified 5% CO_2_ atmosphere in accordance with experimental protocol. Fluorescence intensity was measured 40 min after adding stimuli using a CLARIOstar microplate reader with excitation and emission wavelengths of 488 and 535 nm, respectively.

### 2.5. Nitric Oxide (NO) Detection

NO production was monitored by measuring green fluorescence of triazolofluorescein (DAF-2T), the reaction product of DAF-2 with N_2_O_3_. According to the manufacture protocol, PMNLs were incubated with 5 µM DAF-2 DA for 60 min at room temperature, washed with PBS and resuspended in D-PBS containing 1 mg/mL glucose for at least 30 min before use. Loaded PMNLs were then resuspended in HBSS/HEPES, divided into fibrinogen-coated wells of a 96-well plate (4 × 10^5^ cells/100 µL per well) and incubated at 37 °C, in a humidified 5% CO_2_ atmosphere in accordance with experimental protocol. Fluorescence intensity was measured 40 min after adding stimuli using a CLARIOstar microplate reader with excitation and emission wavelengths of 488 and 535 nm, respectively.

### 2.6. Phagocytosis Assessment

PMNLs (10^6^ cells in 1 mL aliquots of HBSS/HEPES) supplemented or not with FPS-ZM1 were incubated in Eppendorf tubes for 5 min at 37 °C in a humidified 5% CO_2_ atmosphere with continuous stirring. Then, opsonized FITC-labeled *S. typhimurium* bacteria were added so that their multiplicity of infection (MOI) was approximately 20 for further 10 min incubation under the same conditions. PMNLs were then collected by centrifugation at 200× *g*, 4 °C and resuspended in cold PBS followed by flow cytometry (excitation of 488 nm, emission of 525 nm) on a SinoCyte X Flow Cytometer (Biosino Medical Technology Co., Ltd., Suzhou City, China). To differentiate between internalized and surface-bound bacteria, trypan blue (TB), at a working concentration of 1 mg/mL, was used for quenching surface FITC fluorescence [28]. Both relative amounts of PMNLs involved in phagocytosis and fluorescence intensities reflecting an average number of bacteria captured per cell were estimated. Flow cytometry data were analyzed using FlowJo software.

### 2.7. PMNLs Adhesion Assessment

The percentage of firmly attached cells was determined by measuring the absorption of 2,3-diaminophenazine, the colored product of MPO-catalyzed oxidation of o-phenylenediamine dihydrochloride (OPD) by H_2_O_2_ [29]. PMNLs were resuspended in HBSS/HEPES, divided into fibrinogen-coated wells of a F-bottom 96-well plate (2 × 10^5^ cells/100 µL per well) and incubated for 25 min at 37 °C in a humidified 5% CO_2_ atmosphere in accordance with experimental protocol. After removal of non-adherent cells by gently washing them twice with warm PBS, the reaction mixture (4 mM H_2_O_2_ and 5.5 mM OPD in permeabilization buffer: 67 mM Na_2_HPO_4_, 35 mM citric acid, 0.1% Triton X-100, pH 5.0) was added to each well for 5 min at room temperature. The reaction was stopped by adding an equal volume of 1 M H_2_SO_4_, and absorbance at 490 nm was measured with a microplate reader CLARIOstar. A standard curve generated from known numbers of neutrophils was used to convert absorbance values to cell numbers; data are expressed as the percentage of input cells.

### 2.8. Scanning Electron Microscopy (SEM)

PMNLs were resuspended in pre-warmed HBSS/HEPES at a density of 1 × 10^6^ cells/mL and seeded onto fibrinogen-coated glass coverslips placed in 6-well plates (2 mL/well). After 25 min incubation at 37 °C in a humidified 5% CO_2_ atmosphere according to the experimental protocol, adherent cells were fixed with 2.5% glutaraldehyde in HBSS/HEPES supplemented with serine protease and metalloproteinase inhibitors (0.5 mM PMSF and 5 mM EDTA, respectively) for 30 min at room temperature. After washing with buffer, cells were post-fixed with 1% osmium tetroxide in 0.1 M sodium cacodylate buffer containing 0.1 M sucrose (pH 7.3) for 30 min at room temperature. Samples were dehydrated through a graded acetone series (10–100%), dried by critical point dryer Balzers CPD 030 (BAL-TEC GmbH, Schalksmühle, Germany), using liquid CO_2_ as the transition fluid, and sputter-coated with gold/palladium. Imaging was performed using a CamScan S-2 scanning electron microscope (Cambridge Instruments, Cambridge, UK) at an accelerating voltage of 15 kV. Image analysis was performed using ImageJ software version 1.54p (National Institutes of Health, Bethesda, MD, USA).

### 2.9. sRAGE Detection in Cell Culture Supernatants by ELISA

Quantitative assessment of sRAGE in PMNLs supernatants was performed using a commercial ELISA kit. Briefly, PMNLs (10^6^ cells/1 mL HBSS/HEPES per probe) were seeded in fibrinogen-coated 24-well plates and incubated at 37 °C, in a humidified 5% CO_2_ atmosphere in accordance with experimental protocol. After the required incubation time, the supernatants were collected, the cells were precipitated by centrifugation at 200× *g*, 10 min, 4 °C, and the supernatant was centrifuged again at 10,000× *g*, 20 min, 4 °C, and diluted threefold for analysis. Subsequent manipulations were carried out in strict accordance with manufacturer’s instructions. Optical density (absorbance at 450 nm (Abs_450_)) was measured using a CLARIOstar microplate reader. Abs_450_ values were converted into sRAGE concentrations using sRAGE standard curve.

### 2.10. Analysis of RAGE Cellular Localization by Immunofluorescence Microscopy

The methodological recommendations described by Allen L.A. [30] were used for immunofluorescent staining of cells. PMNLs (10^6^ cells/1 mL HBSS/HEPES) were seeded on fibrinogen-coated coverslips placed in Petri dishes and incubated for no more than 5 min at 37 C, 5% CO_2_. The supernatants were then carefully removed followed by fixation (cold 2.5% paraformaldehyde PFA) in PBS, containing 0.5% BSA, 10 min, 4 °C. After removing the fixative solution and washing, samples intended for intracellular antigen staining were permeabilized with ice-cold acetone (3 min) and then rehydrated with PBS. All samples were then blocked for 1 h with 1% BSA/PBS, incubated overnight at 4 °C with Anti-RAGE Recombinant Mouse Monoclonal Antibody [2A11] (1:100 dilution in 0.1% BSA/PBS), and rinsed thoroughly with blocking solution, followed by staining with Goat anti-Mouse IgG Superclonal Recombinant Secondary Antibody, Alexa Fluor 488 (1:1500 dilution in 0.1% BSA/PBS) for 4 h at 4 °C. Nuclei were stained with 0.2 μg/mL Hoechst 33342. The samples were visualized by Olympus IX83 fluorescence microscope (Tokyo, Japan, provided by the Moscow State University Development Program).

### 2.11. Immunofluorescent Staining of Surface and Intracellular RAGE for Flow Cytometry

PMNLs (1 × 10^6^ cells/sample), either freshly isolated or activated according to the experimental protocol, were fixed with 2.5% paraformaldehyde (PFA) in PBS for 15 min at room temperature and washed once with PBS. Cells designated for detection of surface (membrane) RAGE were immediately incubated in blocking buffer (PBS containing 10% normal goat serum) for 45 min at room temperature. For detection of total (surface + intracellular) RAGE, cells were permeabilized with 0.1% Triton X-100 in PBS for 5 min at room temperature prior to blocking, then washed once and blocked as described above.

After blocking, cells were pelleted (300× *g*, 5 min, 4 °C) and resuspended in staining buffer containing either Anti-RAGE Recombinant Mouse Monoclonal Antibody [2A11], diluted 1:100 in staining buffer, Mouse IgG1 Isotype control (MOPC-21) at the same concentration, or staining buffer alone (autofluorescence control). Cells were incubated for 1 h at room temperature in the dark, then washed once with excess PBS.

*Pre-adsorption of secondary antibodies.* To minimize non-specific binding to neutrophil Fc receptors and other cellular components, fluorophore-conjugated secondary antibodies were pre-adsorbed on fixed, permeabilized neutrophils. Briefly, PMNLs (2 × 10^7^ cells) were fixed with 2.5% PFA, permeabilized with 0.1% Triton X-100, washed, and blocked as described above. Goat anti-mouse IgG secondary antibody, Alexa Fluor 488 conjugate, was added at a 1:50 dilution in staining buffer and incubated for 30 min at room temperature with gentle mixing. The cell suspension was then centrifuged sequentially at 200× *g* for 5 min (to pellet cells) and the resulting supernatant at 10,000× *g* for 10 min (to remove residual debris). The cleared supernatant containing pre-adsorbed secondary antibody was collected and stored at 4 °C until use.

Pre-adsorbed secondary antibody was diluted 1:20 in staining buffer and added to cells that had been pre-incubated with either primary anti-RAGE antibody or isotype-matched control antibody. Samples were incubated for 30 min at room temperature in the dark, then washed once with excess PBS and resuspended in PBS for flow cytometry analysis. Autofluorescence control samples (cells incubated with staining buffer alone, without primary or secondary antibody) were processed in parallel to establish baseline fluorescence levels. Cells were analyzed immediately on a SinoCyte flow cytometer. Mean fluorescence intensity (MFI; Ex 488 nm/Em 525 nm) was recorded for 20,000 events per sample after gating on singlets and excluding debris based on forward/side scatter profiles. Flow cytometry data were analyzed using FlowJo software (v10.4.0; BD Biosciences, Ashland, OR, USA), and results were visualized with GraphPad Prism.

### 2.12. Detection of RAGE in Cell Lysates and Supernatants by SDS-PAGE, Western Blot, and Dot Blot

*Sample preparation.* For intracellular RAGE, PMNLs (2 × 10^6^/sample) were pelleted, lysed directly in Laemmli buffer (62.5 mM Tris-HCl pH 6.8, 2% SDS, 10% glycerol, 50 mM DTT, 0.01% bromophenol blue), denatured at 95 °C for 5 min, and analyzed by SDS-PAGE or stored at −20 °C.

For extracellular RAGE, PMNLs (5 × 10^6^/sample in HBSS/HEPES) were stimulated in fibrinogen-coated 6-well plates. Cell-free supernatants were collected with protease inhibitors (1 mM PMSF, 10 µM E64, 5 mM EDTA), cleared by sequential centrifugation (300× *g*, 5 min; then 10,000× *g*, 10 min; 4 °C), and concentrated by acetone precipitation (3:1, −20 °C, overnight). Pellets were washed twice with ice-cold acetone, air-dried, resuspended in SDS-DTT buffer (50 mM Tris-HCl pH 6.8, 2% SDS, 50 mM DTT), mixed 1:1 with 2× Laemmli buffer, denatured, and analyzed by Western or dot blot.

*SDS-PAGE and Western blotting.* Samples were separated on 12% polyacrylamide gels using Mini-PROTEAN Tetra Cell system (Bio-Rad Laboratories, Hercules, CA, USA). Proteins were transferred to PVDF membranes (Thermo Scientific, Rockfold, IL, USA, 0.2 µm) by wet transfer in Towbin buffer (25 mM Tris, 192 mM glycine, 15% methanol, 0.05% SDS) at 100 V for 90 min at 4 °C using a Mini Trans-Blot^®^ Cell, Bio-Rad.

Membranes were blocked with 5% non-fat dry milk in TBS-T (from here on: 20 mM Tris-HCl pH 7.6, 137 mM NaCl, 0.05% Tween-20) for 1 h at room temperature, then probed with primary Anti-RAGE Recombinant Mouse Monoclonal Antibody [2A11] (1:20,000 in 1% non-fat dry milk in TBS-T) overnight at 4 °C and with HRP-conjugated secondary antibody (1:5000) for 1 h at room temperature. After washing 3 × 10 min with TBS-T, membranes were incubated with Rabbit anti-Mouse IgG [H+L] Secondary Antibody, HRP (1:5000 in 1% non-fat dry milk in TBS-T) for 1 h at room temperature. Signals were visualized using Clarity Western ECL Substrate (Bio-Rad Laboratories) and imaged on a ChemiDoc Touch Imaging System (Bio-Rad Laboratories).

*Dot blot analysis.* Denatured supernatant samples (2 µL) were spotted onto PVDF membranes pre-wetted in methanol and equilibrated in TBS. Membranes were air-dried for 30 min, then blocked and probed with primary and secondary antibodies as described for Western blotting.

Image processing and densitometric analysis were performed using Image Lab Software (v5.0; Bio-Rad Laboratories).

### 2.13. Statistical Analysis

Statistical analyses and graphical representations were performed using GraphPad Prism 8.4.3 software (Dotmatics, Boston, MA, USA). To check the normality of distribution before the parametric analysis of the data, the Shapiro–Wilk test was used. A *p*-value of <0.05 was considered statistically significant. RM One-way ANOVA followed by Dunnett’s multiple comparison test was used to quantify data on [Ca^2+^]-flux, phagocytosis, sRAGE secretion and flow-cytometric RAGE expression. Two-way ANOVA followed by Sidak’s (for between groups differences) or Dunnett’s (for within groups differences) tests were used to quantify data on ROS/RNS assessment. An unpaired *t* test was used to quantify data on PMNLs morphology.

## 3. Results

### 3.1. RAGE Inhibition Suppresses Ca^2+^-Dependent ROS Production and iNOS Activation in Neutrophils During Bacterial Recognition

**Figure 1:**
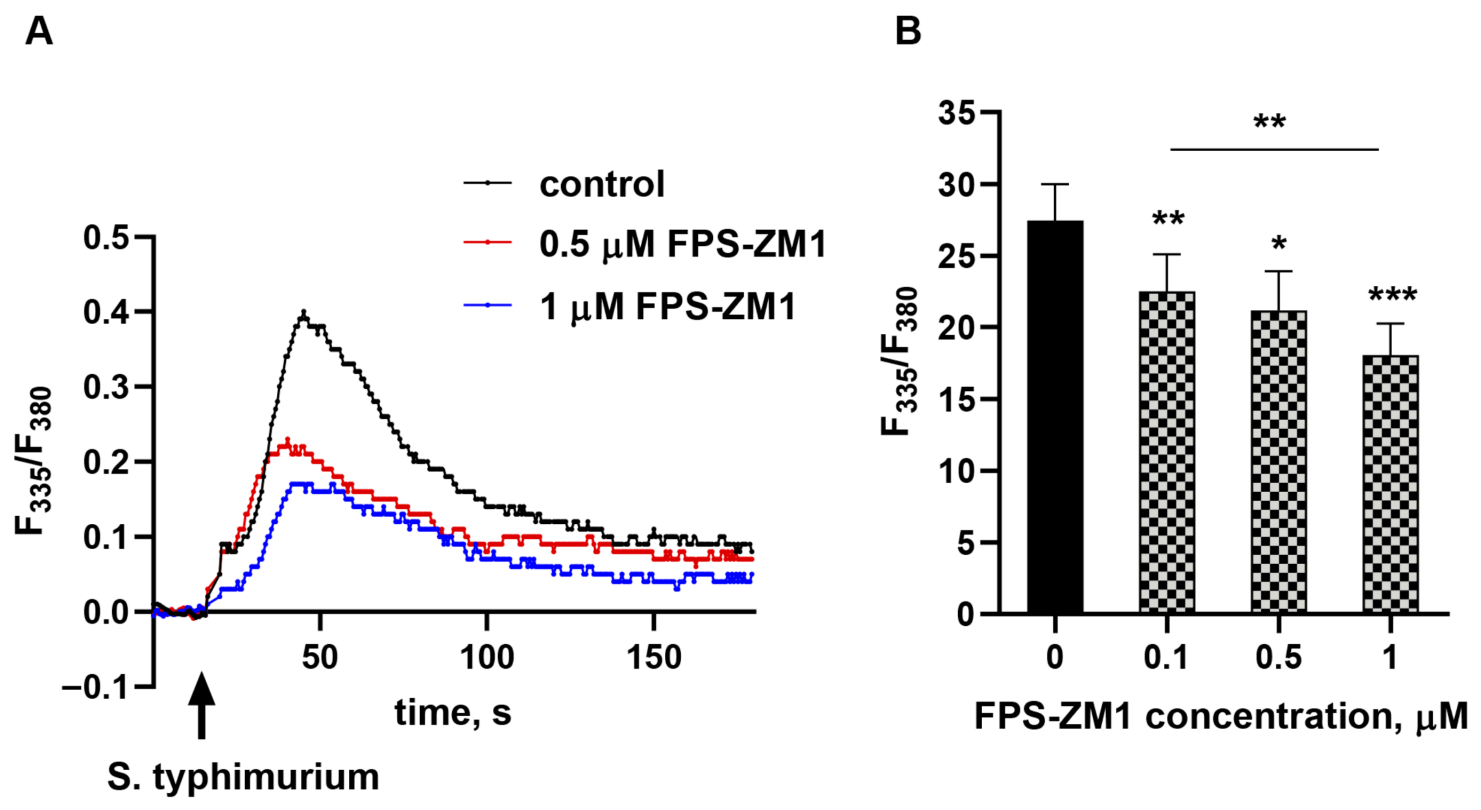
Inhibition of RAGE suppresses the transient increase in neutrophil cytoplasmic Ca^2+^ concentration induced by cell–bacteria interaction. Fura-2 AM-loaded PMNLs were pre-incubated for 5 min without additives (control (**A**) or zero FPS-ZM1 concentration (**B**)) or with FPS-ZM1 in concentrations indicated. Then non-opsonized *S. typhimurium* bacteria (MOI ~20) were injected automatically. Fluorescence intensities (335 nm/510 nm and 380 nm/510 nm) began to be recorded before bacteria injection, and measurements continued for 3 min after. (**A**) Typical curves of [Ca^2+^]_i_ changes (ratio F_335_/F_380_) after adding bacteria to untreated (black) and pre-treated with FPS-ZM1 in indicated concentrations (red, blue) PMNLs. (**B**) AUC (means ± SE) for a three-minutes interval after bacteria addition to untreated (solid black) or pre-incubated with FPS-ZM1 in concentrations indicated (black and gray) PMNLs. * *p* < 0.05, ** *p* < 0.01, *** *p* < 0.001 for pairs of data indicated or compared to control (zero FPS-ZM1) value; *n* = 3.

Neutrophil interaction with a pathogen or soluble stimulus almost always results in a transient increase in cytosolic calcium ion concentration. Ca^2+^ acts as a universal intracellular messenger, essential for the initiation of the vast majority of effector functions of these cells. Treatment of cells with the RAGE inhibitor FPS-ZM1 immediately before addition of bacteria dose-dependently reduces the amplitude of the [Ca^2+^] spike (Figure 1).

**Figure 2:**
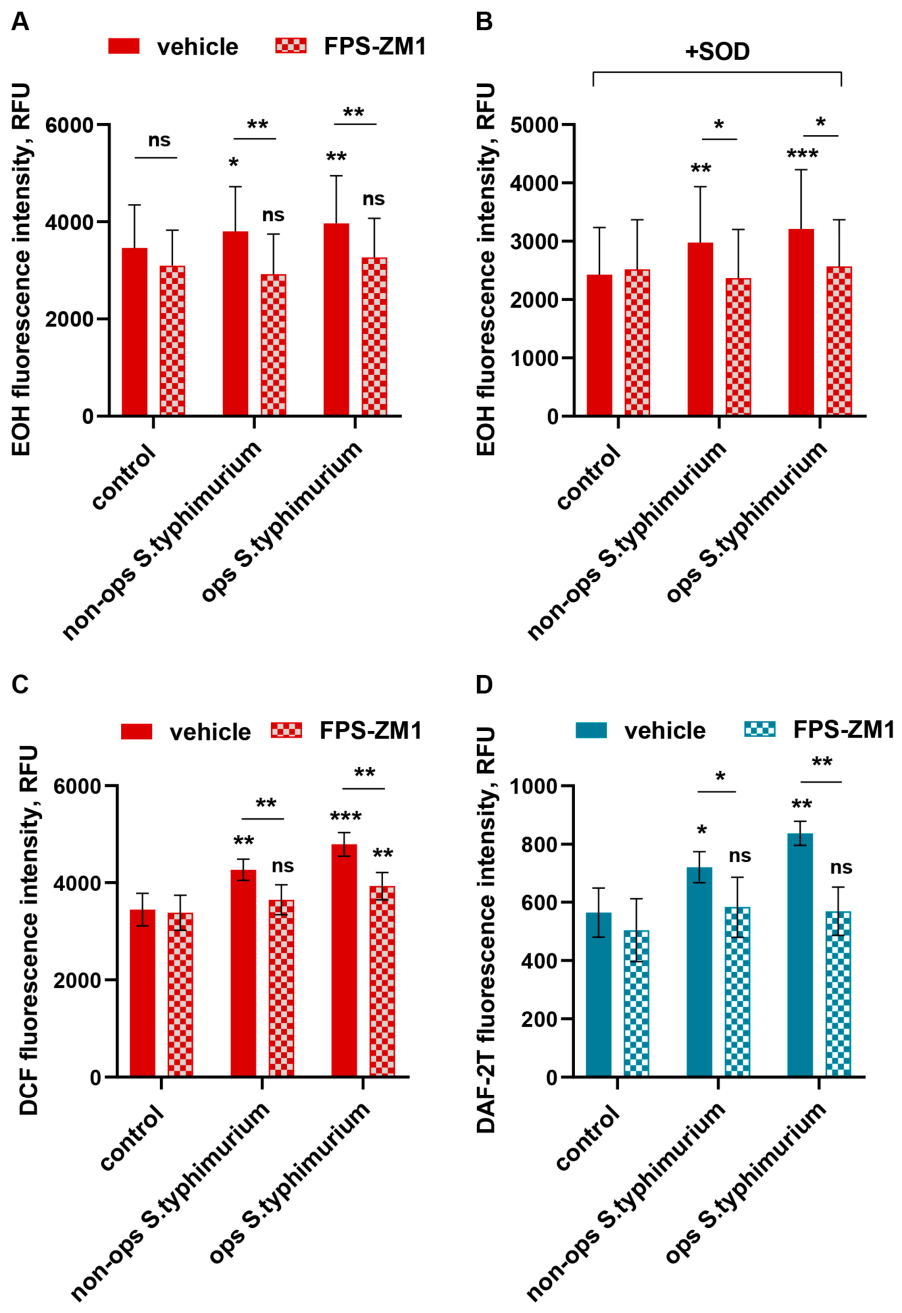
Inhibition of RAGE effectively prevents the formation of reactive oxygen and nitrogen species in neutrophils interacting with bacteria. DHE-stained without (**A**) or with SOD (**B**), or H_2_DCF-DA-loaded (**C**), or DAF-2 DA-loaded (**D**) PMNLs were pre-incubated for 5 min without additives (solid pattern) or in the presence of 1 µM FPS-ZM1 (mesh pattern). Then non-opsonized or opsonized *S. typhimurium* bacteria (MOI ~20) were added to all probes except control ones. Presented are means ± SE of fluorescence intensity values measured at least three independent experiments performed in triplicates. * *p* < 0.05, ** *p* < 0.01, *** *p* < 0.001 for pairs of data indicated or compared to corresponding control values. ns: not significant; *n* = 5.

Elevated calcium levels are critical for the assembly of the multiprotein NADPH oxidase complex, both in the plasma and granule/phagosomal membranes [31], which plays a key role in the granulocyte response to infection. Activation of NADPH oxidase in response to neutrophil contact with a pathogen, or in response to soluble stimuli, leads to the formation of large quantities of superoxide anion, which is converted into more aggressive forms of ROS. Using dihydroethidium, we showed that blocking RAGE with a specific inhibitor suppresses superoxide production (both total and intracellular, as assessed in the presence of SOD) by neutrophils interacting with bacteria (Figure 2A,B). This also leads to a decrease in the pool of intracellular oxidants, which is detected by the non-selective fluorescent probe H_2_DCFH-DA (Figure 2C). The RAGE inhibition effect was present for both non-opsonized and opsonized with donor serum bacteria, indicating the involvement of this receptor in the mechanisms of both “non-immune” (with a predominant role of pattern recognition receptors) and “immune” (principally mediated by opsonins) pathogen recognition. 2,7-Dichlorodihydrofluorescein oxidation occurs not only due to superoxide-anion by-products (e.g., hydroxyl and peroxy radicals) but also by reactive nitric oxide derivatives: •NO and ONOO- [32]. Indeed, the NADPH oxidase inhibitor DPI completely neutralized the prooxidant effects detected using DHE (Supplementary Figure S2A) and only partially reduced DCF fluorescence changes (Supplementary Figure S2B). Neutrophilic granulocytes constitutively express inducible NO-synthase (iNOS). Nitric oxide, abundantly produced by neutrophils during their interaction with bacteria and bacterial metabolic products, particularly with lipopolysaccharides, performs both microbicidal and regulatory functions. iNOS activation, unlike NADPH oxidase, is not a calcium-dependent process [33]. However, by recording the fluorescence of triazolofluorescein, which strictly correlates with nitric oxide synthase activity (Supplementary Figure S2C), we showed that inhibition of RAGE prevents the activation of neutrophil iNOS in response to the addition of bacteria (Figure 2D).

### 3.2. RAGE Inhibition Impairs Neutrophil Phagocytosis and Spreading Without Affecting Primary Adhesion to Fibrinogen

**Figure 3:**
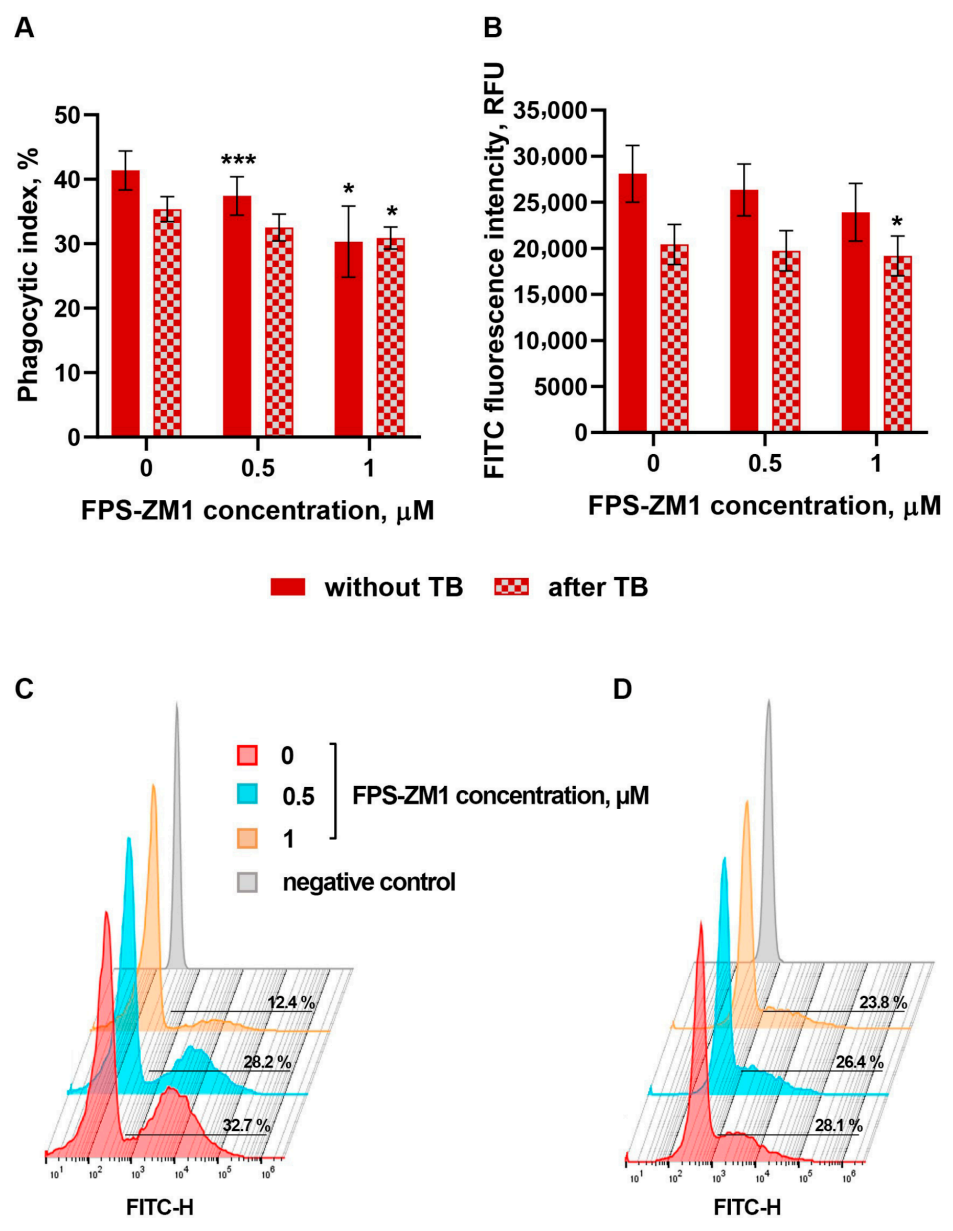
The RAGE receptor is involved in the implementation of phagocytic activity of neutrophils. PMNLs were pre-incubated without additives or with FPS-ZM1 followed by opsonized FITC-labeled bacteria *S. typhimurium* addition for 10 min. Samples with unstained bacteria were used as negative controls. Presented are pooled flow cytometry data (means ± SE) with numbers of phagocytizing (i.e., FITC-positive cells), as a percentage of the total number of PMNLs in the sample (**A**) and fluorescence intensities of FITC-positive subpopulations (**B**) without (solid pattern) or after (mesh pattern) TB quenching. * *p* < 0.05, *** *p* < 0.001 compared to corresponding control values; N = 5. (**C**,**D**) Typical FITC-fluorescence histograms of phagocytizing PMNLs without (**C**) or after (**D**) TB quenching.

Being disassembled in resting PMNLs the cytosolic NADPH oxidase components are targeted to plasma or phagosomal membranes at the moment of action of a soluble stimulus or during phagocytosis. The RAGE receptor plays a significant role in the implementation of phagocytic activity of non-professional phagocytes [34]. Achouiti et al. demonstrated a significant reduction in the phagocytic capacity of peripheral neutrophils isolated from RAGE^−/−^ knockout mice [35]. We assessed how the phagocytic activity of human neutrophils changes under conditions of RAGE inhibition. Short-term pretreatment of cells with FPS-ZM1 reduces both the number of phagocytic neutrophils in the population (Figure 3A,C) and the mean number of bacterial particles per phagocyte (Figure 3B,D), affecting mainly the initial stage and the pathogen attachment to the plasma membrane (Figure 3B, without TB).

**Figure 4:**
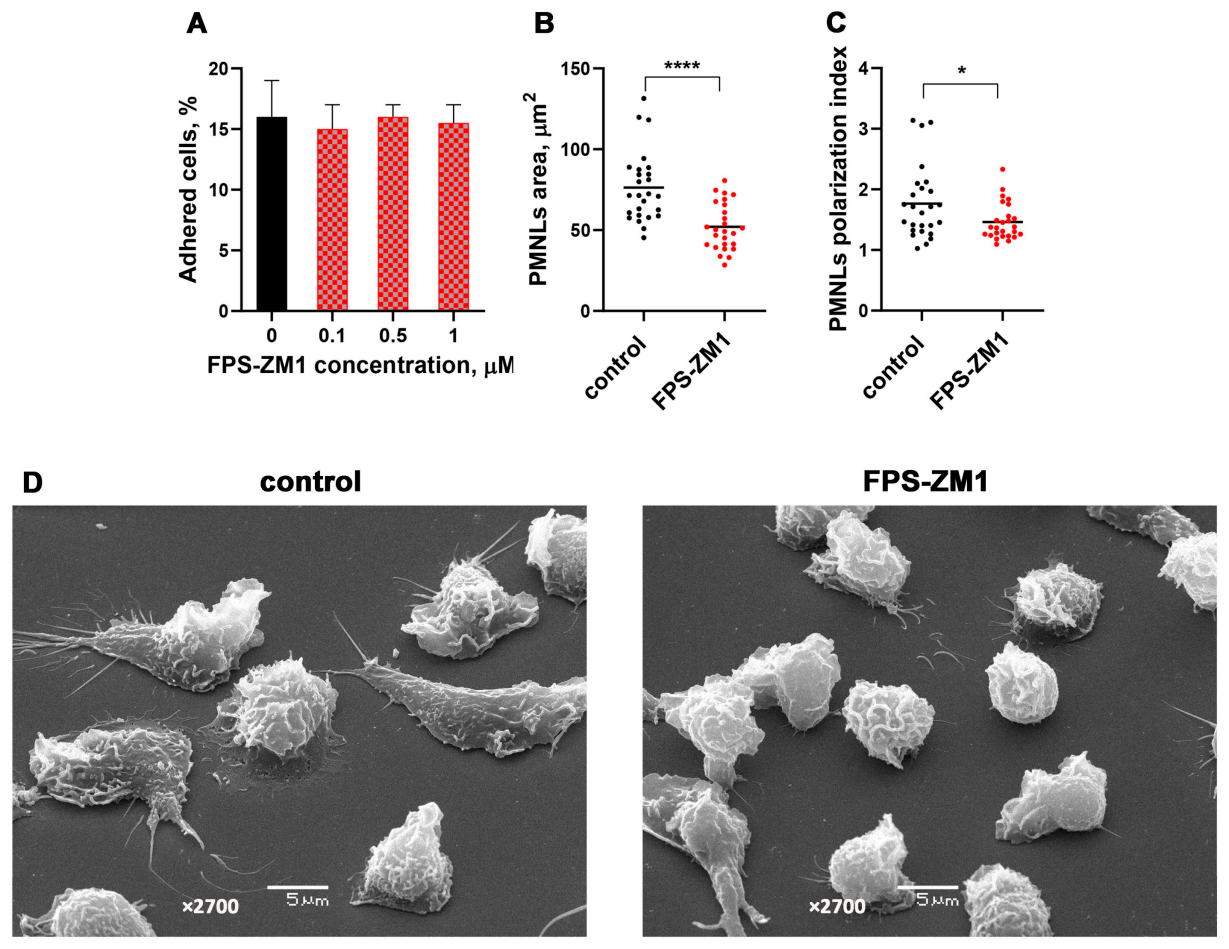
RAGE inhibition impairs neutrophil spreading and polarization on fibrinogen without affecting cell adhesion. (**A**) PMNLs were seeded onto fibrinogen-coated 96-well plates in medium alone (zero) or medium containing the indicated concentrations of FPS-ZM1, and incubated for 25 min. After washing to remove non-adherent cells, adherent neutrophils were quantified using a myeloperoxidase-coupled OPD assay. Data represent mean ± SE of cell adhesion (% of input) from three independent experiments. (**B**–**D**) PMNLs were seeded onto fibrinogen-coated coverslips and incubated for 25 min in the absence (control) or presence of 1 µM FPS-ZM1. Adherent cells were fixed and imaged by scanning electron microscopy; morphometric analysis was performed using ImageJ. Scatter plots show cell area (**B**) and polarization index (**C**) (* *p* < 0.05, **** *p* < 0.0001, n ≥ 25 cells per condition). (**D**) Representative SEM micrographs from three independent experiments. Scale bars: 5 µm.

On a fibrinogen-coated substrate, quantitative assessment of neutrophil adhesion along with SEM imaging of attached cells revealed that RAGE blockade did not alter the fraction of adherent neutrophils (Figure 4A) but significantly suppressed their spreading (Figure 4B,D) and polarization (Figure 4C,D).

Although neutrophils are traditionally viewed primarily as producers of RAGE ligands, our data establish that the receptor itself is essential for both their morphological activation and effector responses to bacterial challenge: RAGE blockade impaired Ca^2+^ mobilization, oxidative and nitrosative burst, and phagocytosis. To understand how RAGE exerts such strong effects, we next investigated its cellular availability by assessing constitutive surface expression, intracellular compartmentalization, and activation-dependent release, aiming to determine whether functional receptor pools are dynamically regulated upon stimulation or constitutively displayed on the membrane.

### 3.3. Activation-Dependent Release of sRAGE from an Intracellular Pool in Neutrophils

**Figure 5:**
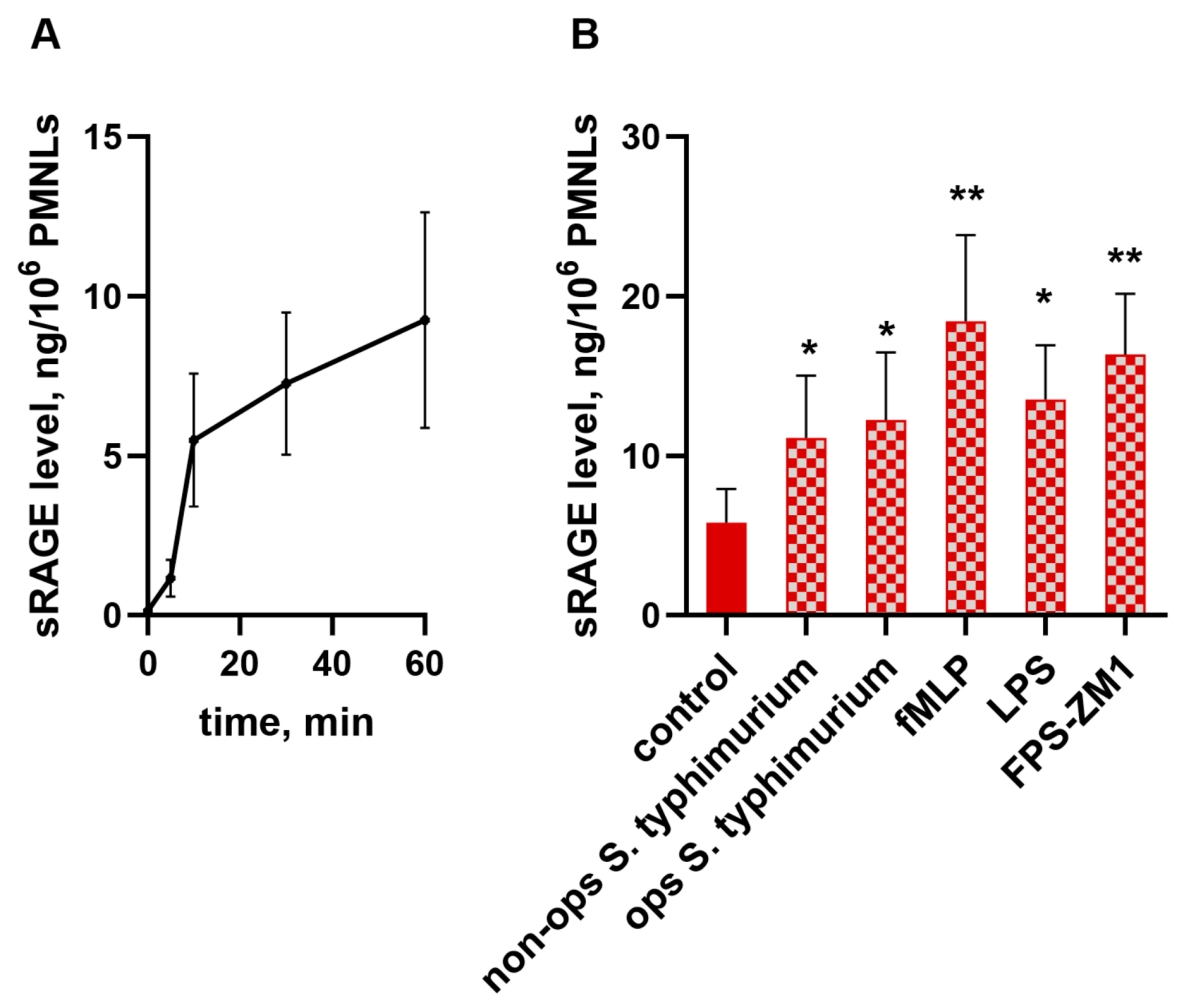
Neutrophil adhesion to a fibrinogen-coated substrate, by itself and in the presence of stimuli, is accompanied by intense release of sRAGE into the extracellular space. PMNLs were incubated for desired period of time (5–60 min) (**A**) or 30 min (**B**) in fibrinogen-coated wells without additives (**A**; **B**—control) or in the presence of non-opsonized or opsonized *S. typhimurium* (MOI~20), 0.01 µM fMLP, 100 ng/mL LPS or 1 µM FPS-ZM1 (as indicated on **B**). At the end of the incubation, the concentration of sRAGE in the supernatants was determined by ELISA. The dynamics of the increase in the concentration of free RAGE in untreated cells over time is presented on (**A**). Average values (means ± SE) of sRAGE content in PMNLs supernatants after 30 min of cultivation are presented on (**B**) (* *p* < 0.05, ** *p* < 0.01 compared to control value, N = 5).

We have previously shown that interaction with *S. typhimurium* bacteria dramatically increases transcription of the RAGE protein in neutrophils [36]. Moreover, it turned out that cell attachment to a fibrinogen-coated substrate is accompanied by the accumulation of sRAGE in the pericellular environment (Figure 5). The kinetic curve of sRAGE release in control samples indicates the process to be most intensive in the first 10 min of incubation (Figure 5A).

**Figure 6:**
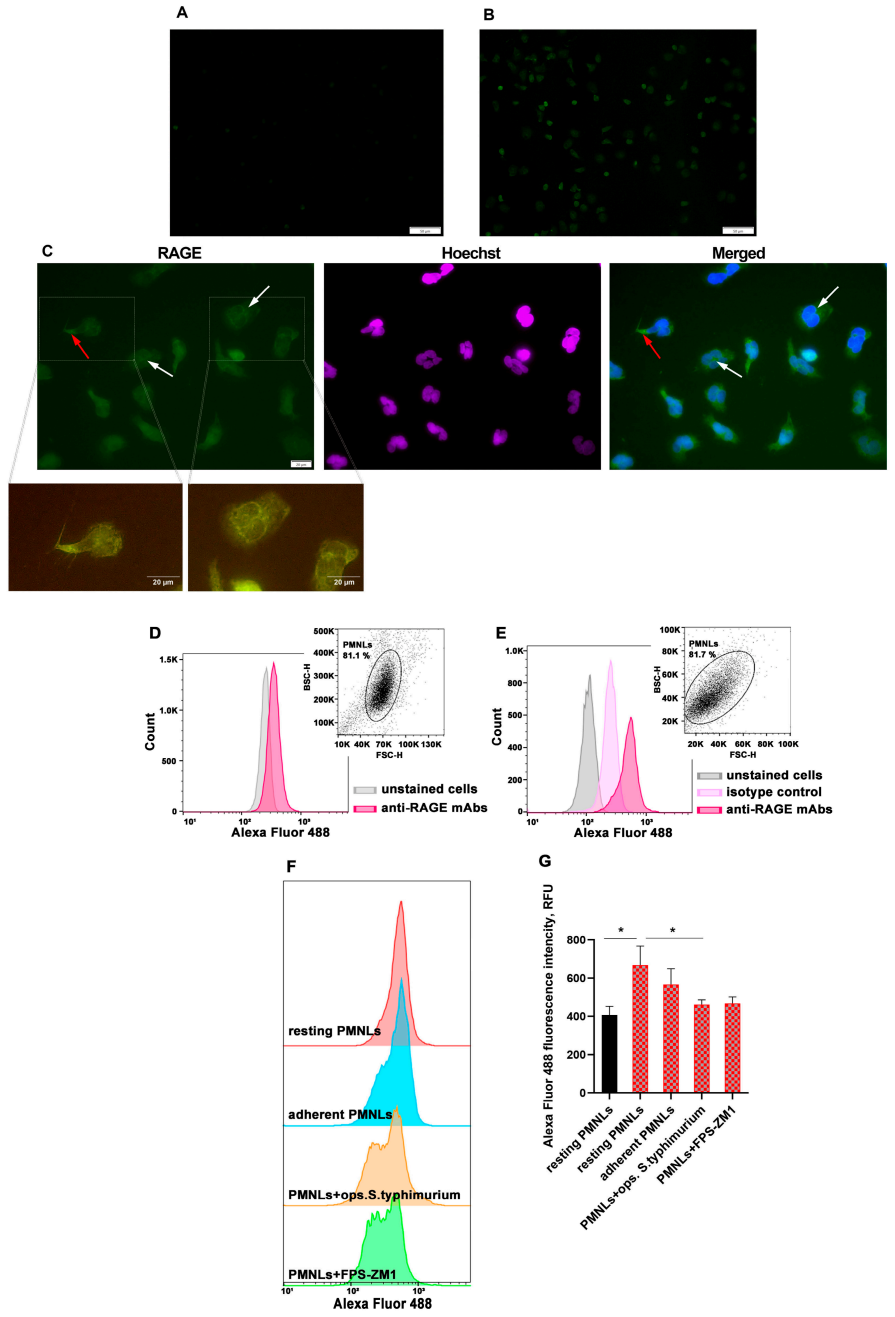
Minimal surface expression, abundant intracellular pool, and activation-dependent release of RAGE in human neutrophils. (**A**–**E**) Resting PMNLs were stained for RAGE under non-permeabilized (surface) (**A**,**D**) and permeabilized (total) (**B**,**C**,**E**) conditions; activated cells were assessed for changes in cell-bound RAGE (**F**,**G**). (**A**–**C**) Representative immunofluorescence images show negligible RAGE signal on intact resting cells (**A**), which increases markedly after permeabilization (**B**,**C**) (RAGE—green, nuclei—blue). (**D**–**G**) Flow cytometry histograms of RAGE immunofluorescence. (**D**) Non-permeabilized resting PMNLs stained for surface RAGE (purple fill) compared to autofluorescence (gray fill). (**E**) Permeabilized resting PMNLs stained for total intracellular RAGE (purple fill) compared to autofluorescence (gray fill) and isotype control (pink fill). (**F**,**G**) Resting PMNLs or cells adhered to fibrinogen-coated surfaces for 30 min in the absence (adherent PMNLs) or presence of *S. typhimurium* (MOI~20) or 1 µM FPS-ZM1 were permeabilized (except “resting PMNLs” sample presented in black on (**G**) and stained for RAGE. Presented are typical fluorescence histograms (**F**) and average fluorescence intensity values (means ± SE, * *p* < 0.05 for pairs of data indicated, N = 3) (**G**).

The addition of bacteria, as well as soluble bacterial-derived stimuli—lipopolysaccharide and the chemotactic peptide fMLP—stimulates sRAGE release. A surprising finding was the ability of the FPS-ZM1 inhibitor we used in this study to dramatically enhance the formation of an extracellular sRAGE pool (Figure 5B). There are two main pathways for the formation of soluble RAGE: shedding by membrane receptor metalloproteinases and formation by alternative splicing. To determine which mechanism is more likely to occur in neutrophils, RAGE protein was visualized using immunofluorescence microscopy. In resting neutrophils (cultured for no more than 5 min on a fibrinogen-coated substrate), membrane RAGE expression was extremely low (Figure 6A). However, membrane permeabilization allows visualization of large amounts of the antigen localized within the cell (Figure 6B,C), primarily in the perinuclear space (Figure 6C, white arrows), most likely in the endoplasmic reticulum located there, as well as RAGE accumulation in areas of cell adhesion to the substrate (Figure 6C, red arrows). Quantitative assessment of cellular RAGE expression by flow cytometry confirmed the microscopy results. Immunofluorescent staining of non-permeabilized (intact) neutrophils revealed RAGE-specific signal at the level of autofluorescence (Figure 6D), confirming negligible constitutive surface expression. Upon permeabilization, the mean fluorescence intensity (MFI) increased approximately 1.5-fold (Figure 6E,G), indicating the presence of a substantial intracellular RAGE pool. Consistent with the ELISA data described above, which demonstrated activation-dependent release of soluble RAGE into the extracellular space (Figure 5), flow cytometry revealed that activation of neutrophils—induced by adhesion to fibrinogen, interaction with bacteria, or treatment with FPS-ZM1—resulted in a reduction in intracellular fluorescence intensity. Consistent with the release of soluble RAGE into the extracellular space, activation of neutrophils—induced by adhesion to fibrinogen, interaction with bacteria, or treatment with FPS-ZM1—resulted in a significant reduction in intracellular fluorescence intensity (Figure 6F,G).

### 3.4. Molecular Forms of RAGE in Neutrophil Lysates and Supernatants

**Figure 7:**
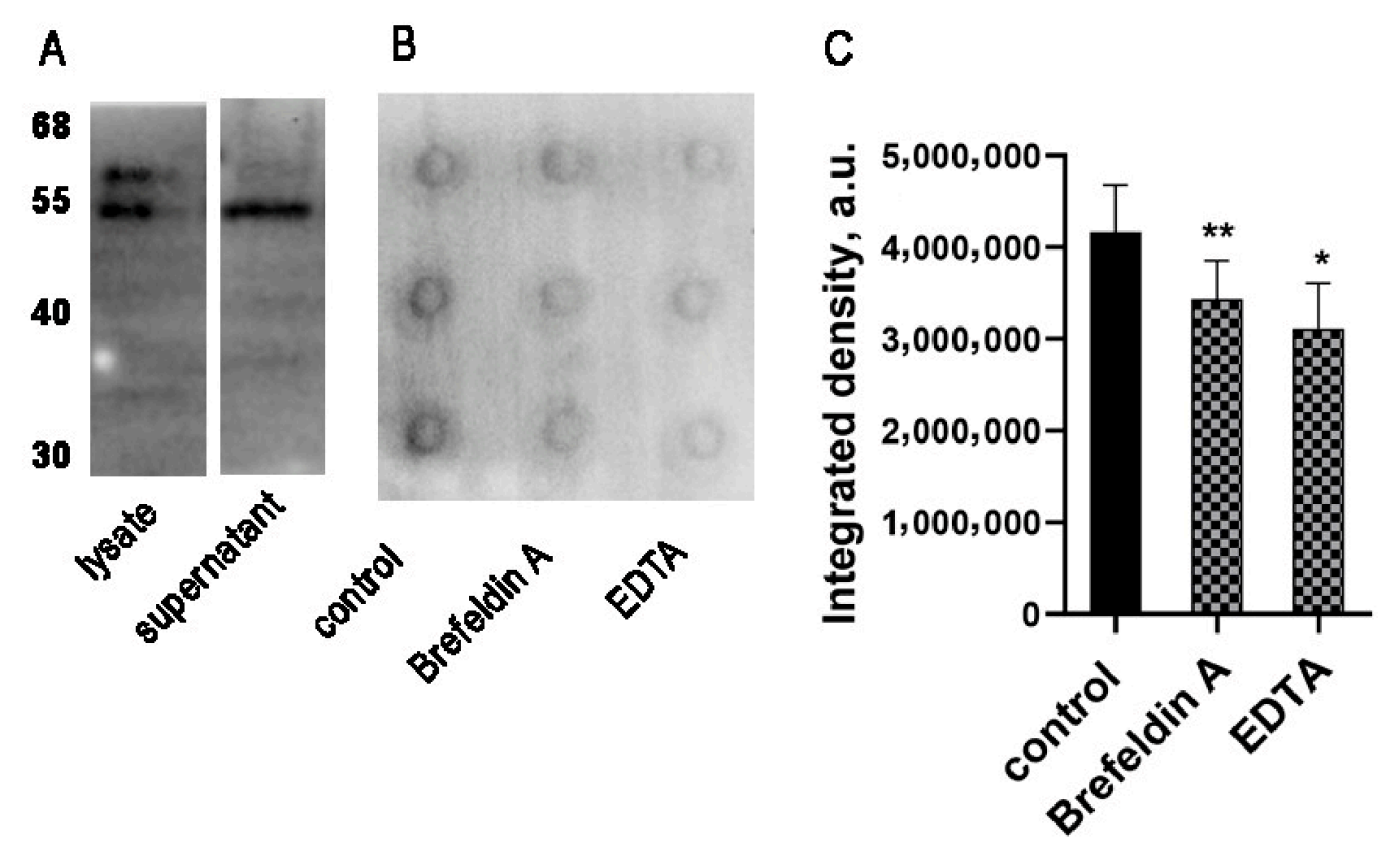
Molecular characterization and regulated release of extracellular RAGE in neutrophils. (**A**) Western blot analysis of RAGE in lysates of resting PMNLs and supernatants of activated neutrophils. (**B**,**C**) Quantification of extracellular RAGE in supernatants from three independent donors after Brefeldin A (3 µg/mL) or 5 mM EDTA treatment vs. control. Shown are a representative dot blot image (**B**) and mean ± SE values (**C**) (* *p* < 0.05, ** *p* < 0.01).

To characterize the molecular nature of intracellular and extracellular RAGE, we analyzed neutrophil lysates and supernatants by Western blotting. In lysates of resting neutrophils, two prominent bands at approximately 55 kDa were detected, along with a weaker band at ~35 kDa (Figure 7A). The ~55 kDa species likely correspond to differentially glycosylated forms of full-length membrane RAGE [37], whereas the ~35 kDa band is consistent with the expected size of endogenously spliced esRAGE [38] or a proteolytic fragment retained intracellularly. Notably, in supernatants of activated neutrophils, the ~35 kDa band was absent, while both ~55 kDa bands were readily detected (Figure 7B), indicating that extracellular RAGE predominantly originates from membrane-associated or full-length forms rather than the splice variant.

To assess the mechanisms governing RAGE release, we quantified extracellular RAGE by dot blot in supernatants of adherent neutrophils treated with Brefeldin A (an inhibitor of ER–Golgi transport) or EDTA (a metalloprotease inhibitor). Both compounds significantly reduced the levels of extracellular RAGE compared to vehicle-treated controls (Figure 7B,C), suggesting that regulated secretion via the conventional secretory pathway and metalloprotease-dependent shedding jointly contribute to the generation of extracellular RAGE upon neutrophil activation.

## 4. Discussion

In neutrophils from healthy donors, we found extremely low levels of RAGE at the plasma membrane in the resting state, but our data clearly show that the full-length receptor still plays an important sensory role, at least for the Gram-negative bacteria *Salmonella typhimurium*. The choice of pathogen was dictated by its clinical significance. Indeed, it is one of the four key pathogens causing gastroenteritis, which can be life-threatening for immunocompromised individuals. Pathologies caused by Gram-negative microorganisms are characterized by a more severe course and more often lead to sepsis [39]. In addition, this type of bacteria is characterized by the development of antibiotic resistance, which is why Gram-negative bacteria, including fluoroquinolone-resistant *Salmonella* spp., predominate in the list of priority pathogens, released by WHO in 2017 [40]. Inhibition of RAGE with FPS-ZM1 strongly reduced the rapid rise in cytosolic [Ca^2+^] that normally follows contact with bacteria. This early calcium signal is known to control many neutrophil effector functions, including activation of NADPH oxidase. RAGE can modulate calcium signaling through several mechanisms, including effects on transient receptor potential (TRP) channels that mediate calcium entry across the plasma membrane [41], and these channels are important for neutrophil chemotaxis and inflammatory responses [42]. Because Ca^2+^ depletion abolishes NADPH oxidase activity [43], it is consistent that blocking RAGE leads to a strong decrease in both total and intracellular superoxide production in response to bacteria.

Our data also show that RAGE inhibition suppresses the formation of reactive nitrogen species in neutrophils. Although iNOS activation is considered Ca^2+^-independent since calmodulin already binds at extremely low intracellular Ca^2+^ concentrations [33], FPS-ZM1 effectively prevented the bacteria-triggered increase in DAF-2T fluorescence. A possible explanation may be related both to the DAF-FM biochemical features and the multilayered regulation of iNOS in neutrophils. First, DAF-2 primarily reacts with dinitrogen trioxide (N_2_O_3_), a product of NO aerobic oxidation, rather than free NO itself [44]; thus, its signal is influenced by local O_2_ availability, superoxide dynamics, and the overall cellular redox environment. Given that RAGE blockade attenuates NADPH oxidase-dependent ROS production, the reduced DAF-2 signal may partially reflect diminished N_2_O_3_ formation due to altered redox dynamics. Second, while mature iNOS operates independently of Ca^2+^, its expression is controlled by Ca^2+^-sensitive transcriptional pathways [45] and ROS-dependent signaling cascades [46], both impaired by FPS-ZM1. We therefore propose that RAGE signaling does not directly modulate the enzyme’s catalytic function but rather permits increased iNOS levels and creates a favorable redox environment for detectable RNS formation. This model aligns with the observed coordination between Ca^2+^ flux, oxidative burst, and nitrosative output, and with reports of FPS-ZM1 reducing oxidative damage in other RAGE-dependent pathologies [22,47]. Collectively, these data position RAGE as a central hub that couples pathogen recognition to coordinated ROS/RNS effector responses in neutrophils.

In line with data from RAGE^−/−^ mice, which show reduced phagocytic activity of neutrophils [35], we observed that short-term treatment with FPS-ZM1 decreased both the proportion of phagocytic cells and the number of bacteria internalized per cell. The strongest effect was seen at the stage of bacterial attachment to the neutrophil surface. Attachment defect closely aligns with our morphological findings; although RAGE blockade left baseline adhesion to fibrinogen unchanged, it profoundly suppressed subsequent cell spreading and polarization. These data indicate that RAGE is dispensable for initial integrin-mediated tethering but critically required for the cytoskeletal remodeling that drives phagocytic cup formation and efficient pathogen engagement. The RAGE signaling pathway likely has intersection points with the Rho GTPase (Rac1/Cdc42) and actin-regulatory complexes to coordinate membrane protrusion around the target. Although the precise molecular adaptors linking RAGE to the actin nucleation machinery in neutrophils remain to be fully defined, it is well established that RAGE engagement activates downstream signaling modules, including Src family kinases and MAPKs [48], that are known to modulate Rho GTPase activity in various cell types [49]. Given that Rac1 and Cdc42 are canonical, essential drivers of phagocytic cup formation [50], it is plausible that RAGE blockade impairs these pathways in neutrophils, thereby explaining the observed defects in cell spreading and bacteria attachment. By impairing this morphological activation, FPS-ZM1 not only limits bacterial uptake but also disrupts the spatial assembly of the NADPH oxidase complex on the phagosomal membrane, thereby explaining the concomitant suppression of ROS and RNS production. Collectively, these data position RAGE as a signaling node that couples early pathogen recognition to downstream effector programs through coordinated control of neutrophil shape change and cytoskeletal dynamics.

A key finding of our study is the dynamic regulation and dual behavior of RAGE in neutrophils. On the one hand, we show that full-length RAGE acts as a sensor that supports calcium mobilization, NADPH oxidase activation, NO production, and phagocytosis. On the other hand, we observed rapid accumulation of soluble RAGE (sRAGE) in the extracellular environment when neutrophils adhere to a fibrinogen-coated surface and when they are stimulated with bacteria, LPS, or fMLP. The kinetics of this release, with a peak during the first 10 min, suggest that sRAGE may function as a fast negative-feedback mechanism. By acting in a way that sequesters RAGE ligands (AGEs, S100 proteins, HMGB1) in the extracellular space, as a decoy receptor by binding RAGE ligands in the extracellular space, RAGE could limit further activation of membrane RAGE on both neutrophils and other surrounding cells.

The cellular source and molecular nature of extracellular RAGE appear to be cell type specific. While alveolar epithelial cells generate sRAGE predominantly through proteolytic cleavage of membrane-bound full-length RAGE [51], our data indicate that neutrophils utilize a combined mechanism. Western blot analysis revealed that intracellular RAGE exists both differentially glycosylated ~55 kDa forms (consistent with full-length membrane RAGE) and ~35 kDa species (compatible with endogenously spliced esRAGE). Notably, supernatants of activated neutrophils contained only the ~55 kDa forms, suggesting that the extracellular pool originates primarily from membrane-associated or secreted full-length protein rather than the splice variant. Furthermore, the reduction in extracellular RAGE by both Brefeldin A (an inhibitor of ER–Golgi transport) and EDTA (a metalloprotease inhibitor) implies that regulated secretion of pre-formed intracellular stores and metalloprotease-dependent shedding operate in parallel to generate extracellular sRAGE upon neutrophil activation.

The paradoxical effect of FPS-ZM1 further refines this model. While pharmacological inhibition of RAGE reduces calcium signaling, ROS/RNS production, and phagocytosis, which is consistent with RAGE’s role as a positive signaling hub, it simultaneously amplifies the release of sRAGE. Immunofluorescence microscopy and flow cytometry indicate that resting neutrophils contain a substantial intracellular RAGE pool, concentrated in the perinuclear region and likely corresponding to the endoplasmic reticulum and associated secretory compartments. Upon activation, this intracellular pool diminishes concomitantly with sRAGE accumulation in the supernatant. We propose that FPS-ZM1, by blocking ligand-induced RAGE signaling at the plasma membrane, may trigger a compensatory mobilization of the intracellular RAGE reservoir, either via enhanced constitutive secretion or through feedback upregulation of sheddase activity. This would elevate extracellular sRAGE levels, potentially reinforcing the decoy-receptor brake on inflammation. Alternatively, FPS-ZM1 may alter RAGE trafficking or stability, favoring its diversion from the plasma membrane into secretory or sheddase-accessible pools.

**Scheme 1:**
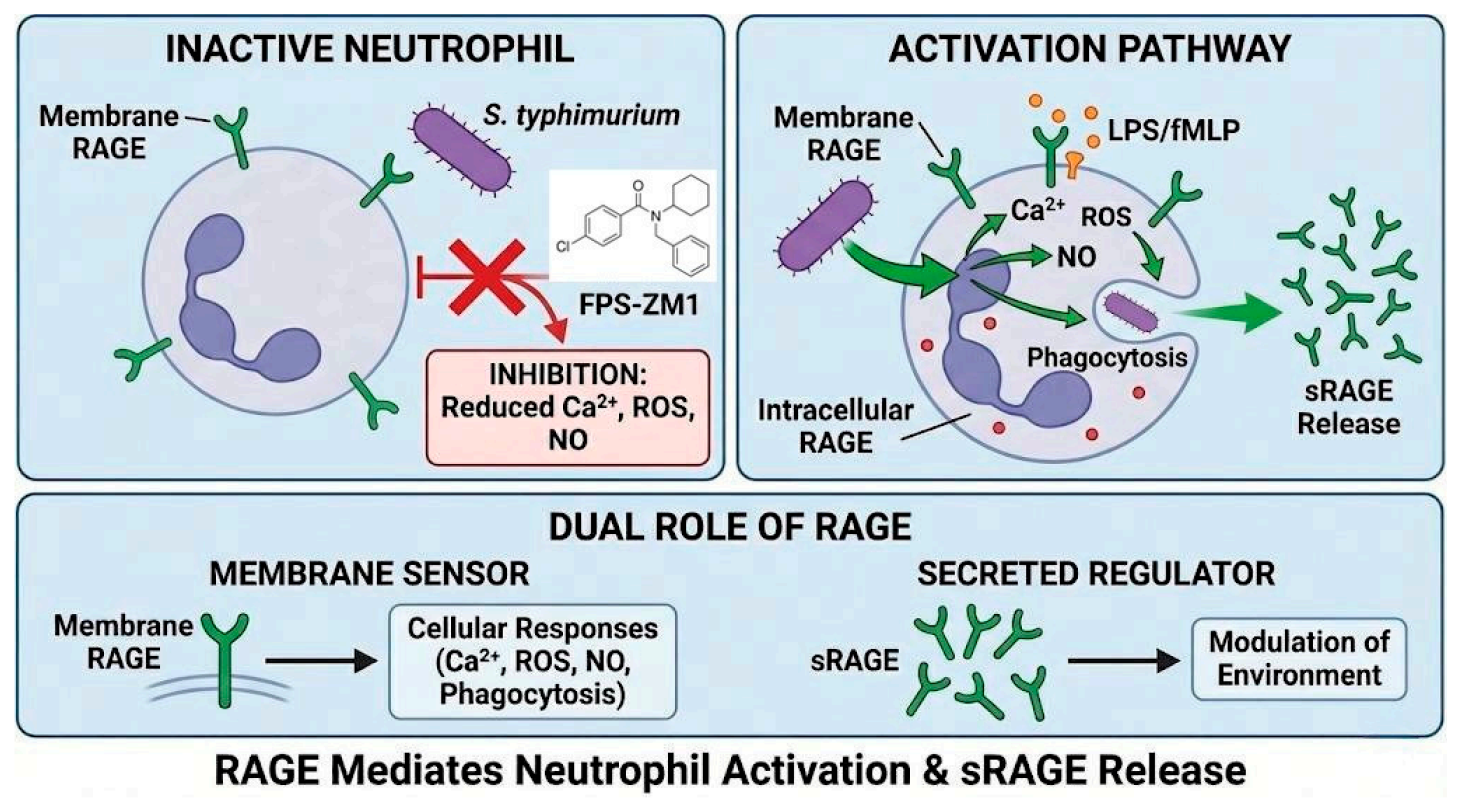
Dual role of RAGE in neutrophil activation by bacteria: both membrane sensor and secreted regulator.

Taken together, our findings support a model in which neutrophil RAGE functions not as a static receptor but as a dynamic, multi-compartmental regulator of innate immunity (Scheme 1). At the plasma membrane, full-length RAGE acts as a pattern-recognition and co-receptor that translates encounters with Gram-negative bacteria into key effector responses—calcium mobilization, oxidative burst, NO/RNS production, and efficient phagocytosis. Concurrently, neutrophils actively generate and release soluble RAGE (sRAGE), which may limit or spatially confine RAGE-dependent signaling by sequestering extracellular ligands and by reducing membrane receptor availability through regulated shedding. This dual capacity—to drive rapid activation while licensing a soluble negative-feedback mediator—suggests a sophisticated self-limiting circuit that helps balance antimicrobial defense with protection of host tissues from excessive oxidative and inflammatory damage. Dysregulation of this equilibrium, whether through excessive ligand exposure, impaired sRAGE release, or pharmacological modulation, could tip neutrophil responses toward either inadequate pathogen clearance or exaggerated tissue injury, with broad implications for acute and chronic inflammatory pathologies. A better understanding of the balance between membrane RAGE and sRAGE in neutrophils, and of how this balance changes in vivo during infection and in chronic diseases, will be essential for the rational design of RAGE-targeted therapies.

## Data Availability

The original contributions presented in this study are included in the article/Supplementary Material. Further inquiries can be directed to the corresponding author.

## Supplementary Materials

The following supporting information can be downloaded at: https://www.mdpi.com/article/10.3390/biomedicines14051128/s1, Supplementary Figure S1: (A,B) Isolated cells were incubated for 30 min at 37 °C in culture dishes, fixed by 2.5 % PFA and stained with 0.2 μg/mL Hoechst. Then both brightfield (demonstrating the integrity of the cells) and fluorescent (demonstrating the characteristic shape of the nuclei of neutrophils) images were obtained on Olympus IX83 fluorescence microscope. C Smears of the isolated cell suspension after fixation with ethanol were colorized according to Romanovsky-Giemsa method. Visible are a purple colouration of nuclei and neutrophil granules. Supplementary Figure S2: DHE- supplemented (A), or H2DCF-DA-loaded (B), or DAF-2 DA-loaded (C) PMNLs were pre-incubated for 5 min without additives (black) or in the presence of NADPH-oxidase (DPI) or NO-synthase (L-NAME) inhibitors (grey). Then non opsonized or opsonized *S. typhimurium* bacteria (MOI~20) were added to all probes except control ones. Presented are means ± SEM of fluorescence intensity values measured in three independent experiments performed in triplicates. ns—non significant, * *p* < 0.05, ** *p* < 0.01, *** *p* < 0.001 compared to corresponding control values.

## Abbreviations

The following abbreviations are used in this manuscript:

RAGE	Receptor for advanced glycation end products
PRR	Pattern-recognition receptor
AGEs	Advanced glycation end products
PMNLs	Polymorphonuclear leukocytes
HMGB1	High mobility group box 1
TLRs	Toll-like receptors
ROS	Reactive oxygen species
RNS	Reactive nitrogen species
fMLP	N-Formyl-L-methionyl-L-Leucyl-L-Phenylalanine
LPS	Lipopolysaccharides
