# Supplementary material for: RAGE in Neutrophils: A Sensor for Pathogen-Associated Structures and Beyond
Source: Biomedicines. 2026 May 16;14(5):1128. doi: 10.3390/biomedicines14051128 (PMC13204100; doi:10.3390/biomedicines14051128)
Supplement: Supplementary file 1 [file biomedicines-14-01128-s001.zip › biomedicines-4293988-supplementary.pdf]

A B C

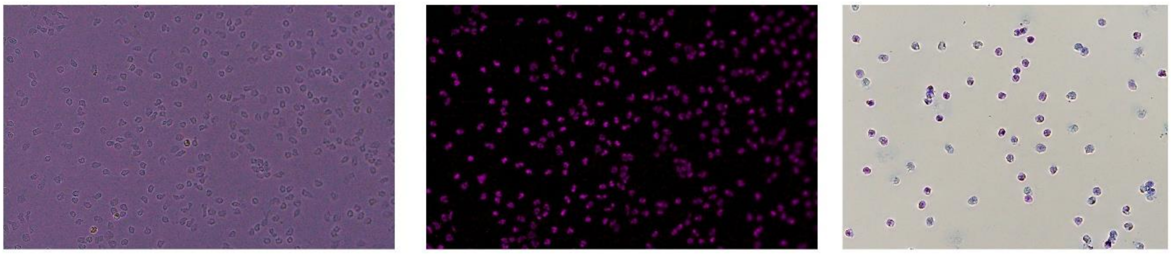

Supplementary Figure S1. A,B Isolated cells were incubated for 30 min at 37 °C in culture dishes, fixed by 2.5 % PFA and stained with 0.2 µg/mL Hoechst. Then both brightfield (demonstrating the integrity of the cells) and fluorescent (demonstrating the characteristic shape of the nuclei of neutrophils) images were obtained on Olympus IX83 fluorescence microscope. C Smears of the isolated cell suspension after fixation with ethanol were colorized according to Romanovsky-Giemsa method. Visible are a purple colouration of nuclei and neutrophil granules.

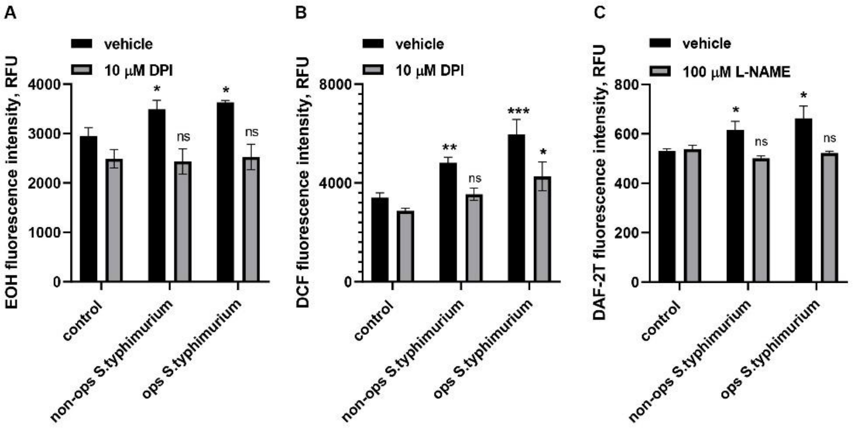

Supplementary Figure S2. DHE- supplemented (A), or H_2DCF-DA-loaded (B), or DAF-2 DA-loaded (C) PMNLs were pre-incubated for 5 min without additives (black) or in the presence of NADPH-oxidase (DPI) or NO-synthase (L-NAME) inhibitors (grey). Then non opsonized or opsonized S. typhimurium bacteria (MOI ~20) were added to all probes except control ones. Presented are fluorescence intensity values measured in three independent experiments performed in triplicates. ns – non significant, *p < 0.05, **p < 0.01, ***p < 0.001 compared to corresponding control values.
